# Model Selection in Systems Biology Depends on Experimental Design

**DOI:** 10.1371/journal.pcbi.1003650

**Published:** 2014-06-12

**Authors:** Daniel Silk, Paul D. W. Kirk, Chris P. Barnes, Tina Toni, Michael P. H. Stumpf

**Affiliations:** Centre for Integrative Systems Biology at Imperial College London, London, United Kingdom; Tum, Germany

## Abstract

Experimental design attempts to maximise the information available for modelling tasks. An optimal experiment allows the inferred models or parameters to be chosen with the highest expected degree of confidence. If the true system is faithfully reproduced by one of the models, the merit of this approach is clear - we simply wish to identify it and the true parameters with the most certainty. However, in the more realistic situation where all models are incorrect or incomplete, the interpretation of model selection outcomes and the role of experimental design needs to be examined more carefully. Using a novel experimental design and model selection framework for stochastic state-space models, we perform high-throughput *in-silico* analyses on families of gene regulatory cascade models, to show that the selected model can depend on the experiment performed. We observe that experimental design thus makes confidence a criterion for model choice, but that this does not necessarily correlate with a model's predictive power or correctness. Finally, in the special case of linear ordinary differential equation (ODE) models, we explore how wrong a model has to be before it influences the conclusions of a model selection analysis.

## Author Summary

Different models of the same process represent distinct hypotheses about reality. These can be decided between within the framework of model selection, where the evidence for each is given by their ability to reproduce a set of experimental data. Even if one of the models is correct, the chances of identifying it can be hindered by the quality of the data, both in terms of its signal to measurement error ratio and the intrinsic discriminatory potential of the experiment undertaken. This potential can be predicted in various ways, and maximising it is one aim of experimental design. In this work we present a computationally efficient method of experimental design for model selection. We exploit the efficiency to consider the implications of the realistic case where all models are more or less incorrect, showing that experiments can be chosen that, considered individually, lead to unequivocal support for opposed hypotheses.

## Introduction

Mathematical models provide a rich framework for biological investigation. Depending upon the questions posed, the relevant existing knowledge and alternative hypotheses may be combined and conveniently encoded, ready for analysis via a wealth of computational techniques. The consequences of each hypothesis can be understood through the model behaviour, and predictions made for experimental validation. Values may be inferred for unknown physical parameters and the actions of unobserved components can be predicted via model simulations. Furthermore, a well-designed modelling study allows conclusions to be probed for their sensitivity to uncertainties in any assumptions made, which themselves are necessarily made explicit.

While the added value of a working model is clear, how to create one is decidedly not. Choosing an appropriate formulation (e.g. mechanistic, phenomenological or empirical), identifying the important components to include (and those that may be safely ignored), and defining the laws of interaction between them remains highly challenging, and requires a combination of experimentation, domain knowledge and, at times, a measure of luck. Even the most sophisticated models will still be subject to an unknown level of inaccuracy – how this affects the modelling process, and in particular experimental design for Bayesian inference, will be the focus of this study.

Both the time and financial cost of generating data, and a growing understanding of the data dependancy of model and parameter identifiability [1], [2], has driven research into experimental design. In essence, experimental design seeks experiments that maximise the expected information content of the data with respect to some modelling task. Recent developments include the work of Liepe et. al [2] that builds upon existing methods [3]–[8], by utilising a sequential approximate Bayesian computation framework to choose the experiment that maximises the expected mutual information between prior and posterior parameter distributions. In so doing, they are able to optimally narrow the resulting posterior parameter or predictive distributions, incorporate preliminary experimental data and provide sensitivity and robustness analyses. In a markedly different approach, Apgar et. al [8] use control theoretic principles to distinguish between competing models; here the favoured model is that which is best able to inform a controller to drive the experimental system through a target trajectory.

**Figure 1:**
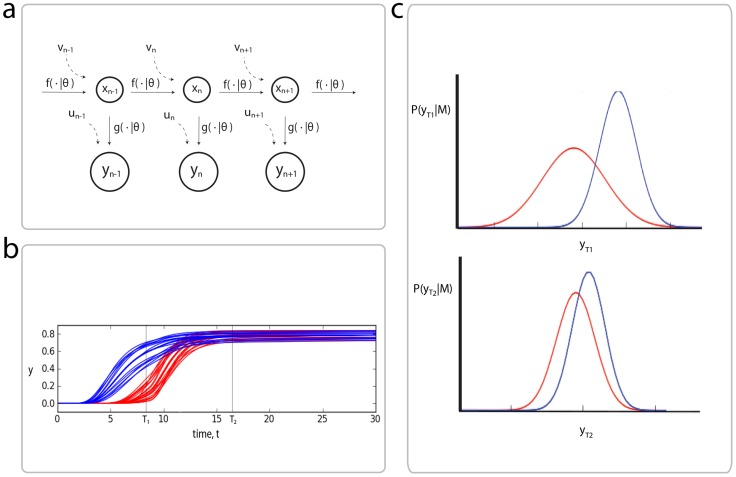
Outline of the proposed experimental design framework. a) We will be concerned with state-space formulations, which model a true state, 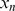, as it evolves under the parametric function 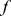 subject to a process noise, 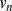, and observations made of this process, 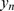, via the 'observation' function, 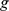, with measurement noise 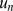. b) Plots of simulations from two different models (blue and red) for various parameter values, under the same experimental conditions. At time 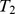, the behaviour of the two models is very similar, while at time 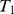, the trajectories separate. c) Gaussian approximations of the model simulations at times 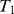 and 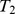 (in general these will be mixtures of Gaussians) obtained via the unscented transform. Time 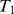 is likely to be more informative than time point 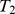 for model selection purposes. Experiments can be scored by how separated these distributions are, which we quantify using the Hellinger distance.

In order to explore the effects of model inaccuracies we work with a computationally efficient experimental design framework. We build on the methods of Flassig and Sundmacher [9] where expected likelihoods are predicted using efficient Sigma-point approximations and leveraged for optimal experimental design, and Busetto et al. [10] where choosing the optimal measurement readouts and time points is undertaken in an iterative fashion, using Sigma-point approximations to update the posterior distributions. Here we show how mixtures distributions may be exploited to cope with non-Gaussian parameter and predictive distributions and further, derive an extension to the case of stochastic state space models. The intuition behind the approach (described fully in Materials and Methods) is shown in Figure 1, where for identical inputs, two ODE models (illustrated in blue and red respectively) are simulated for a range of parameter values, with times 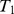 and 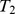 representing two possible choices of times at which the true system can be measured and data gathered. Time 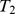 represents an uninformative experimental choice since the behaviour of the two models is very similar, while data obtained at time 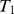 is more likely to favour one model over another, since the distributions of simulated trajectories completely separate. More formally, the key steps in the method are as follow: Firstly we define the limited range of experimental options to be explored and encode them as parameterised extensions of the competing models. Secondly, the so called unscented transform (UT) [11] is used to approximate the prior predictive distribution as a mixture of Gaussians, for each model and a given experiment. Finally, optimisation is performed over the experiment parameters in order to best 'separate' the prior predictive distributions of the competing models. Parameters obtained by this optimisation represent an experiment whose generated data is predicted to maximise the differences in the subsequent marginal likelihood values of the models.

The contributions of this article are threefold; firstly, we extend a promising and computationally efficient experimental design framework for model selection to the stochastic setting, with non-Gaussian prior distributions; secondly, we utilise this efficiency to explore the robustness of model selection outcomes to experimental choices; and finally, we observe that experimental design can give rise to levels of confidence in selected models that may be misleading as a guide to their predictive power or correctness. The latter two points are undertaken via high-throughput *in-silico* analyses (at a scale completely beyond the Monte Carlo based approaches mentioned above) on families of gene regulatory cascade models and various existing models of the JAK STAT pathway.

## Results

### Identifying crosstalk connections between signalling pathways

We first illustrate the experimental design and model selection framework in the context of crosstalk identification. After observing how the choice of experiment can be crucial for a positive model selection outcomes, the example will be used to illustrate and explore the inconsistency of selection between misspecified models.

**Figure 2:**
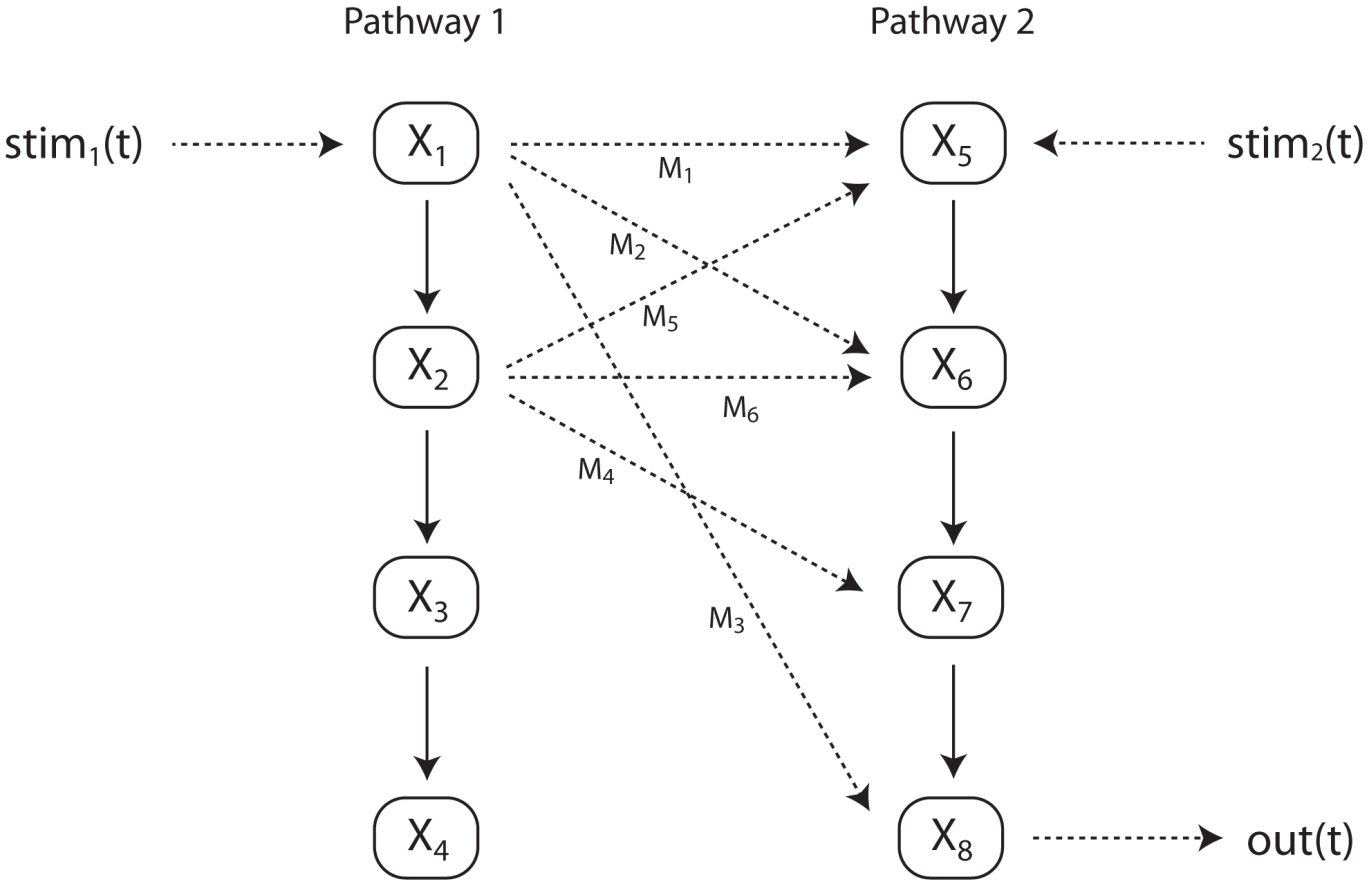
Crosstalk between regulatory cascades. Our task is to identify an unknown crosstalk connection between pathways 1 and 2. A limited range of experiments are considered, involving external stimulation of 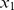 and 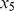, and observation of 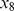, and a set of models (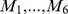) corresponding to different crosstalk options are selected between. The times and strengths of the stimuli, and the time of measurement of 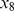 are optimised to best distinguish between the competing crosstalk models.

We consider pairs of regulatory cascades, each consisting of four transcription factors, modelled by ordinary differential equations of the form, 
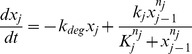
for 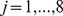, where 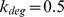 is the rate at which protein 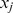 degrades, 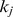 represents the maximal rate of production of 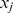, 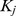 is the amount of the transcription factor, 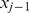, needed for half the maximal response, and 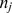 is called the Hill-coefficient, and determines the steepness of the response. A range of crosstalk models are formed (Figure 2) by inserting additional regulatory links between 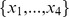 and 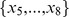 with the same kinetics as above. A single model is chosen as the 'true' biological system to which we perform experiments, and six others with equal prior probabilities are proposed as models of the true system – our task will be to identify the most suitable one.

An experiment is defined by the parameter 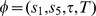, where 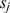 denotes the strength of an external stimulus to the production of 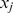, 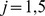 which is modelled as a term, 
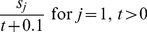
(1)

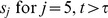
(2)

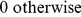
(3)added to the relevant ODE equations. The time delay between the two stimulus applications is given by 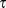, and 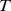 is the time at which a single measurement of the system (of species 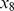 only) is taken. Prior distributions for the model parameters are set as Gaussian with means of 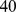 and covariances of 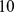 for both the 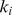 and 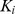 respectively, with the Hill coefficient fixed at 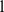.

**Figure 3:**
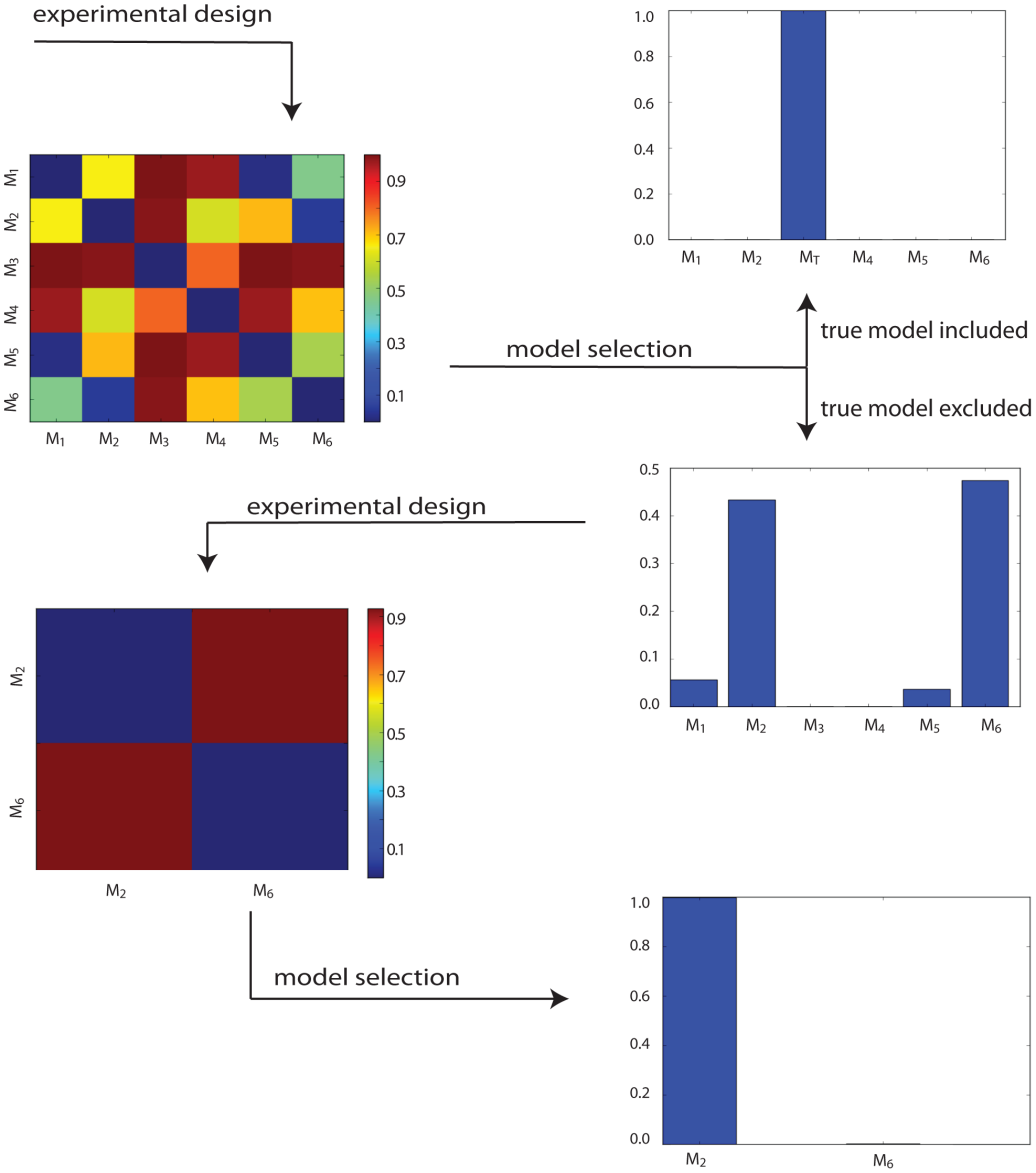
Flow diagram showing two rounds of experimental design and model selection. The heat maps on the left show the Hellinger distances between the prior predictive distributions of model pairs, for the chosen experiments. Bar plots on the right give the posterior probabilities of each model with respect to data produced by the chosen experiment. After the first experiment, models 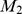 and 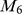 have the most support, but evidence to choose between them is negligible. However a second experiment designed for only these two models (with priors set according to the posterior probability proportions after the first round of model selection) strongly favours model 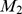.

The results of this round of experimental design are shown in the top left of Figure 3, where a good choice of 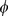 is found to be 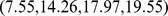, with a corresponding score of 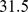. From the figure, it can be seen that this experiment is predicted to distinguish some pairs of models better than others. In particular, the distribution of scores suggests that while the marginal likelihoods of most pairs of models are separated as desired, there is no power to discriminate between models 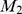 and 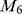, or models 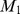 and 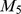. Indeed, data obtained by performing the experiment upon our 'true' system, leads to posterior probabilities for each model with the same pattern.

As a sanity check, we first choose the true model from amongst the set of competing models (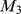), and as expected find that it is recovered by model selection with probability 1. However if the true model is not represented by 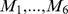 (a far more realistic case) but instead the crosstalk model with a single connection from 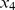 to 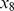, then models 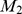 and 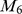 are found to have similar posterior probabilities of approximately 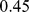. Likewise, 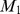 and 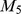 share a posterior probability of 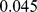, while a clear difference exists between any other pair of models. To distinguish further between the pair of highest scoring models, a further round of experimental design was performed, with the resulting experiment and data providing strong evidence in favour of model 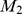.

**Figure 4:**
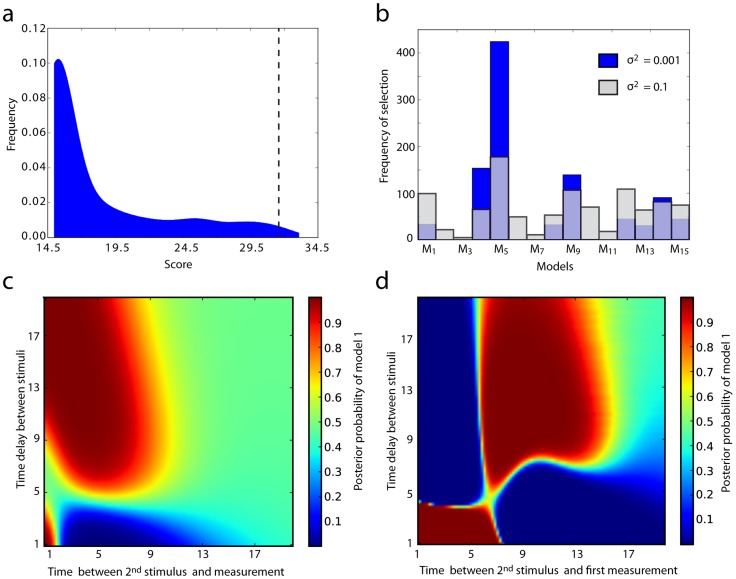
Robustness of model selection. a) Frequency distribution of scores for 1000 uniformly sampled values of 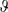. Scores concentrate around the interval 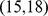, corresponding to very little information content. The dotted line indicates the score of 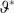 chosen in the first round of experimental design. b) Using the 16 crosstalk models consisting of a single connection from pathway 1 to 2, a true model is fixed and 1000 uniformly sampled experiments are performed upon it. The frequencies at which the remaining 15 crosstalk models are selected, with each data set considered independently are shown. (blue) At a low level of measurement noise (with variance 0.01) model 5 is chosen most frequently, but is still outperformed for over half the experiments. (grey) When the measurement noise is increased to a variance of 0.1, the choice of model becomes even less robust. c, d) Each heatmap shows the posterior probabilities of model 1 (versus model 2), calculated independently for 9025 experiments, with data sets of different sizes (1 and 8 respectively). Each coordinate represents a different experiment, with variations to both the time delay between stimuli, and the measurement times.

In an attempt to evaluate the added value of choosing 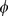 rationally for this example, we calculate scores for a uniform sample of 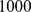 values of 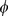 from the same range as explored above. The resulting score distribution shown in Figure 4a, peaks in the interval 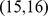 which corresponds to an average Hellinger distance of 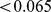 between the maximally separated marginal likelihoods of each pair of models. This is in contrast to the experiment found by our approach which lives in the tail of the distribution, with an average Hellinger distance of 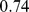, and highlights how unlikely it is to find suitable experiments by chance alone. Experiments with even higher information content are found, which suggests that more care could be taken with the optimisation of 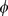, by for example, increasing the population size, or number of generations of the genetic algorithm used.

Perhaps unnervingly, the evidence in the first experiment is found to contradict (though not significantly in this case) the decision in favour of model 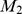 over 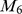, which is based on additional data from the second experiment. This suggests the possibility that the choice of experiment influences not only the amount of information available to select a particular model, but also the outcome of the model selection itself. Indeed the distribution of independently selected models from data generated by random experiments is surprisingly flat (Figure 4b). Even at very low levels of assumed noise, the most frequently selected model is chosen for less than half the experiments undertaken. This has been, to our knowledge, completely overlooked by the experimental design literature, but has important implications that we will explore further below.

### The robustness of model selection to choice of experiment

To examine this last observation in more detail, we work with three of the crosstalk models described above, with connections between, 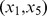, 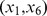 and 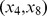 respectively. The last of these is designated as the true model, and the others are considered as competing hypotheses about the location of the crosstalk connection. We perform 36100 experiments to collect data sets of size 1, 2, 4 and 8 equally spaced time points, each consisting of simulating the true model with different values of 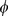 that correspond to changes in the delay between stimulus applications, and variation of the time at which the state of 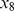 is first measured. An independent round of model selection is performed for each data set, and the posterior probabilities for each model are calculated.

The results for data sets of size 1 and 8 are illustrated in Figure 4c and 4d as heatmaps of posterior probabilities of the first model, and show that the vast majority of the space of experiments is split into distinct regions of high, low and equal probability for each model. In the case of a single time point, most of the explored experiment subspace is found to be uninformative, with the data providing equal support for each model. Three other distinct regions are identified, of which two show decisive support (on the Jeffreys scale) for the first model, and one for which the second model is chosen decisively. In other words, by varying the experimental conditions an unequivocal choice (in isolation) for either model can be obtained. As more data points are considered, the uninformative region grows smaller, but regions of decisive support for each model remain. Interestingly, these regions are located in distinctly different places for single or multiple time points, although they remain similar for 2 or more time points. This reflects the added value of time series experiments – the marginal likelihoods now balance the ability of the models to reproduce each time point, with their ability to capture the autocorrelation of the time series.

**Figure 5:**
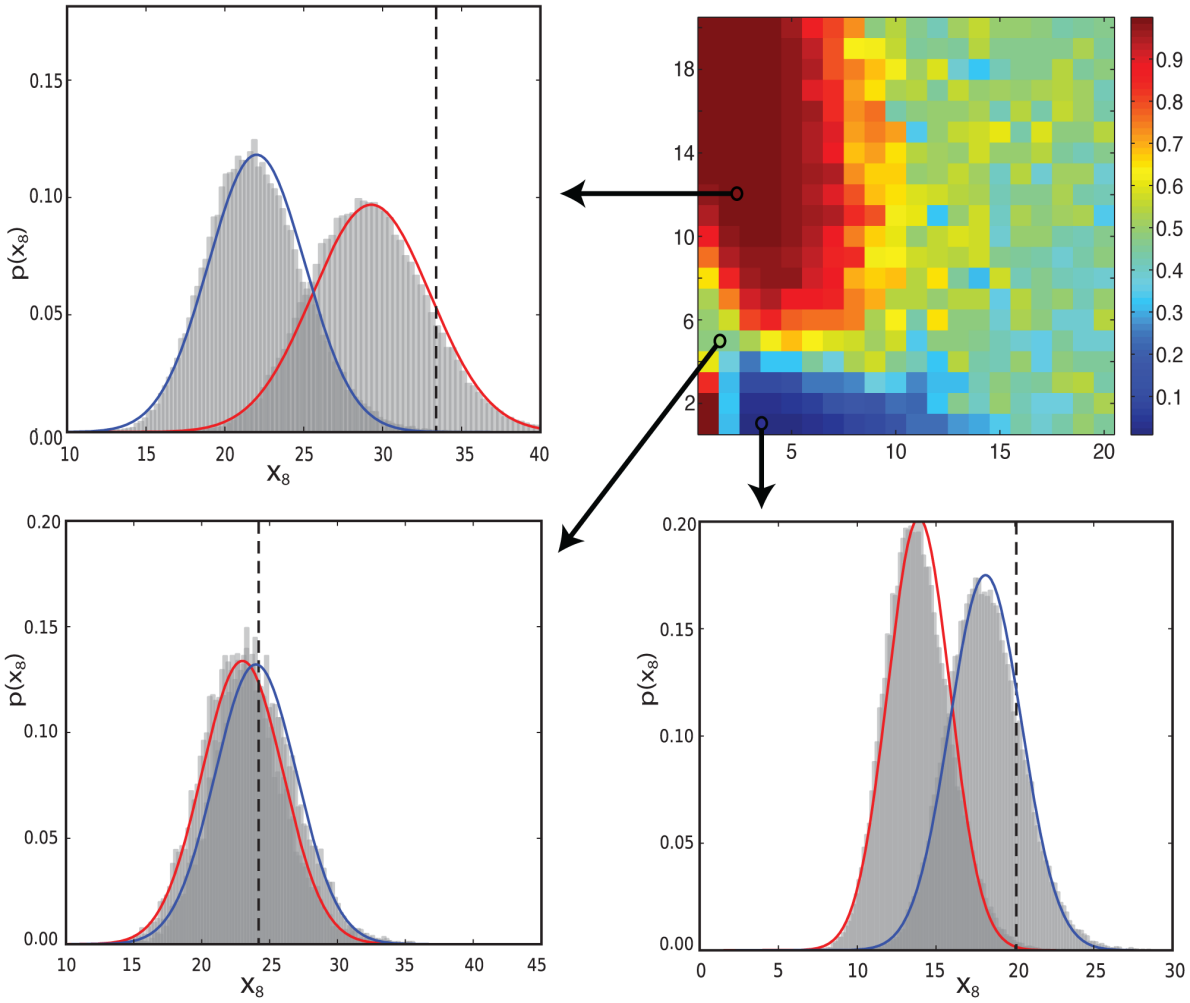
Monte Carlo validation. The top right plot shows posterior model probabilities obtained using MultiNest. The necessarily course grained results match those obtained by the UT in figure 4c. Each of the other plots compare UT approximations to the prior predictive distributions with Monte Carlo approximations using samples of size 10000, for different experimental conditions indicated by arrows. The red and blue lines correspond to UT approximations (using a single Gaussian component) for model 1 and 2 respectively. The dotted line indicates the data simulated from the true model.

In order to establish whether the observed inconsistencies are an artefact of the UT approximations, we perform a similar but necessarily course grained study using MultiNest [12], [13], an implementation of nested sampling (a Monte Carlo based technique with convergence rate 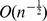 [14]). Results obtained using MultiNest (shown in the upper right of figure 5) are almost identical to those of figure 4c, displaying the same regions of decisive support for each model. Given how difficult it is to estimate marginal likelihoods in general, the excellent performance of the UT (with only one Gaussian component) may seem rather surprising, until one notes that for the models and experiments considered, the prior predictive distributions are approximately Gaussian themselves (Figure 5). We discuss how the framework can deal with non-Gaussian effects, such as those found in the next examples, in the appendix.

### JAK-STAT signalling

**Figure 6:**
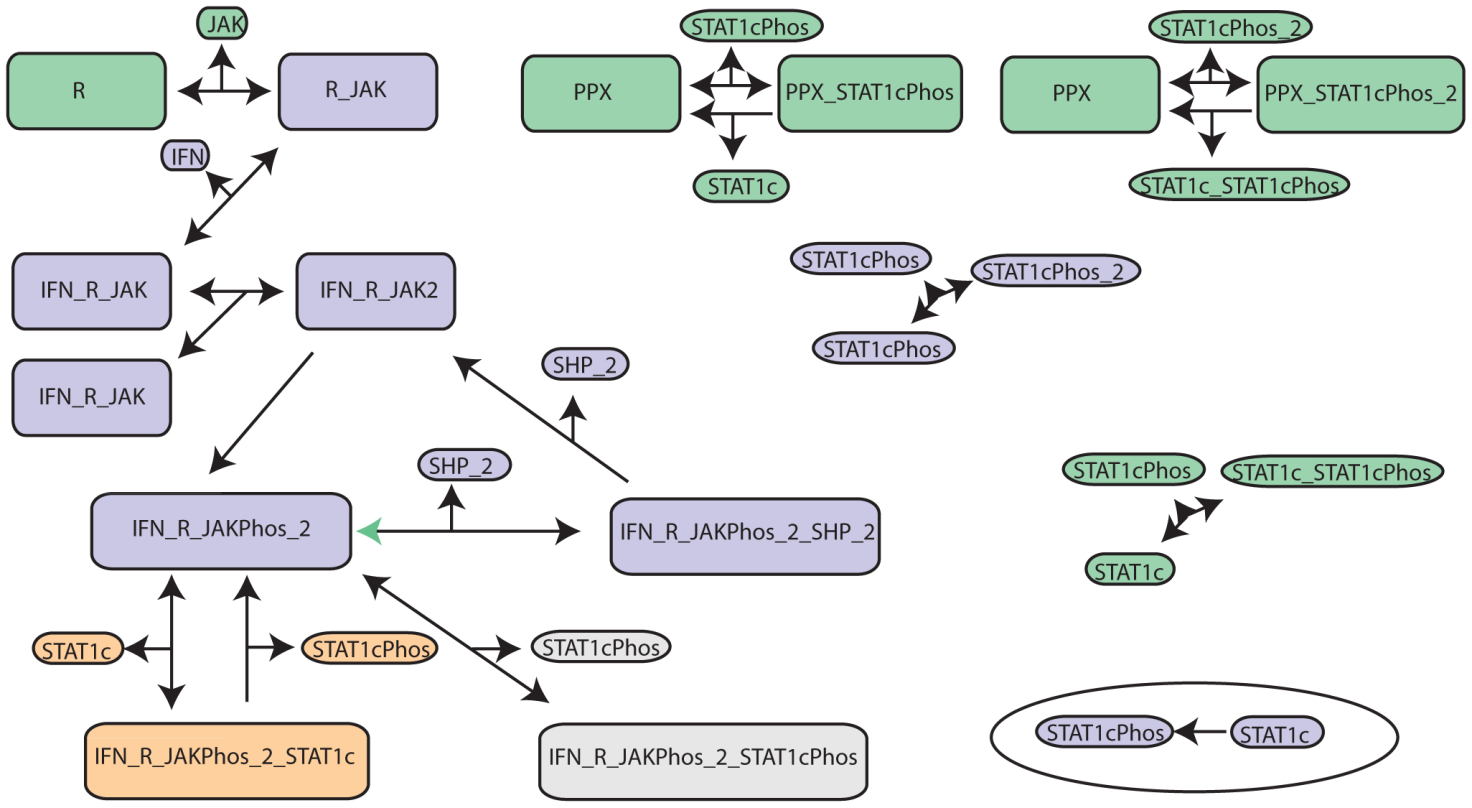
JAK STAT pathway models (adapted from Quaiser et al. [15]). Arrows indicate association or dissociation reactions between the protein species. Grey reactions only occur in the true model, (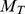). Model 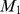 consists of the purple, orange and green components. Model 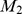 is obtained by removing the green components, and replacing the orange reactions by the reaction in the bottom right oval.

In this section we undertake an analysis of three mass action models of varying degrees of resolution of the JAK-STAT signalling pathway [15]. Each model describes the initial pathway activity after receptor activation (Figure 6), but before any feedback occurs. In brief, the signalling process consists of a receptor binding to JAK to form a complex that can dimerise in the presence of interferon-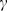 (IFN). This dimer is activated by phosphorylation by JAK, and in turn deactivated after being bound by tyrosine phosphatase (SHP_2). In its active state, the receptor complex phosphorylates cytoplasmic STAT1, which is then able to dimerise and act as a transcription factor [16].

We take the most detailed model, 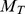, with 17 state variables and 25 parameters (published by Yamada et al. [16]), as our true system to which *in-silico* experiments can be performed, and select between two of the other models proposed by Quaiser et al. The first of these competing models, 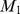, simplifies the true system, by neglecting a reaction – the re-association of phosphorylated STAT1 to the activated receptor – and thereby reducing the system to 16 states and 23 parameters. A series of five other 'biologically inspired' simplifications leads to our second model, 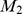, which has 9 states and 10 parameters (these steps are summarised in Figure 6).

We set the parameter priors as a 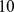 component mixture of Gaussians fit to a uniform sample from the hypercube 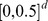, where 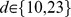 is the parameter dimension, such that all the parameter values inferred for each model by Quaiser et al. are supported. We define and undertake two classes of experiment upon the true model (with parameters fixed to the published values); in the first, the IFN stimulus strength and the initial time point of a time series of 8 equally spaced measurements of the amount of JAK bound to the receptor are varied, and in the second, the species to be measured and the time at which this first measurement takes place are adjusted.

**Figure 7:**
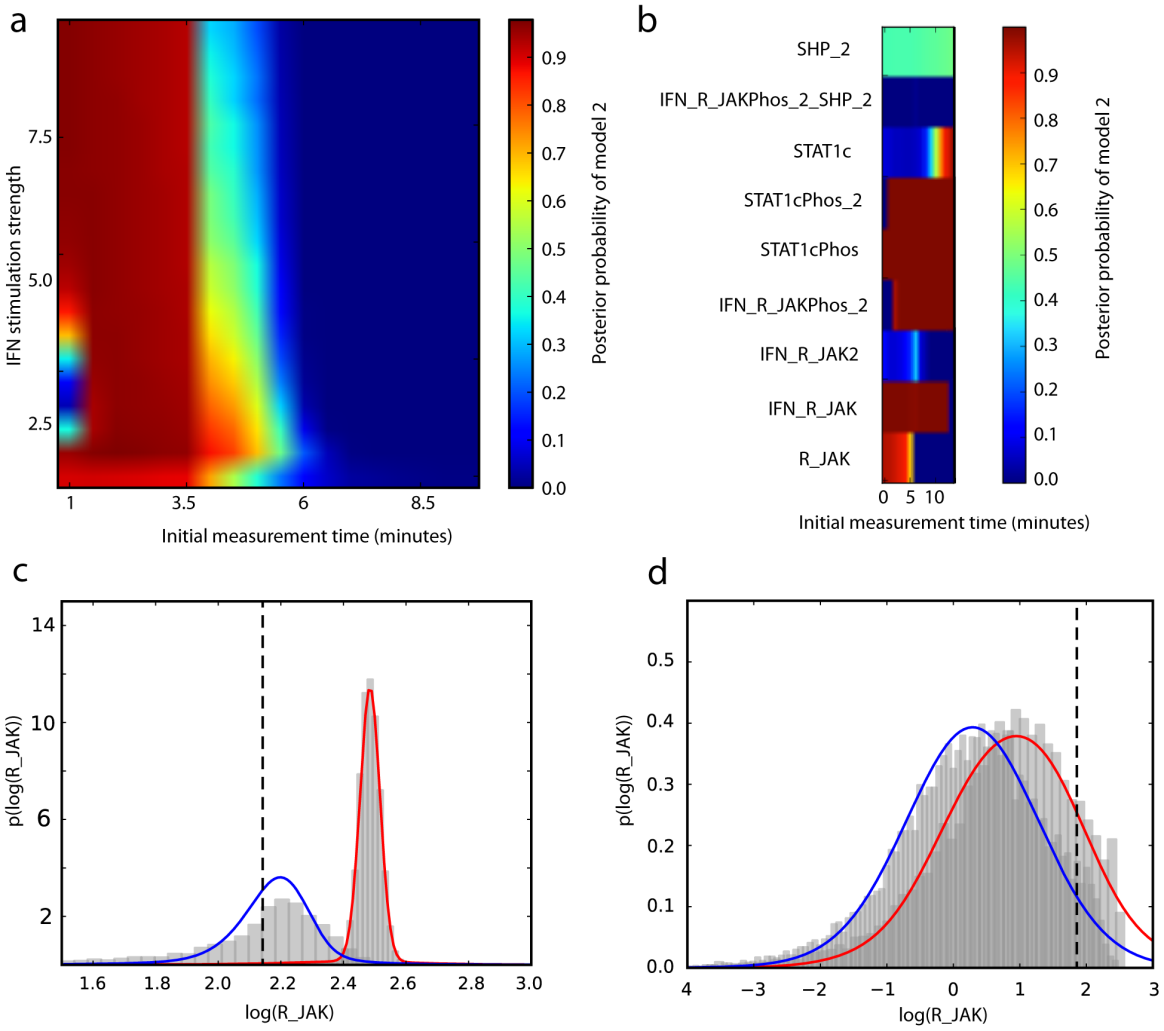
JAK STAT model selection sensitivity. a) IFN stimulus strength and the initial measurement time are varied. b) The species to be measured and the time at which this initial measurement takes place are adjusted. In both figures, distinct regions of high probability for each model can be seen. Comparison of UT and Monte Carlo approximations to the prior predictive distributions when c) 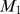 is chosen and d) 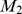 chosen for IFN stimulus strengths 1 and 0.6 at times 60 and 1 minute respectively. The 10 components used in the mixture distribution allow non-Gaussian effects to be captured. The error in the UT approximations is significantly smaller than the differences between models.

Model selection outcomes for each experiment (shown in Figure 7) show similar features to those for the crosstalk models, with distinct region of high posterior probability for each model. For the first class of experiments, selection between models 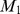 and 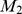 reveals strong support for the simpler model when data is gathered at earlier time points. The more complex model, 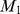, is generally favoured for later time series, and also for a very limited range of IFN stimuli strengths at early time series. For the second class of experiments, the model selection outcome is found to depend strongly upon which species is measured. The simpler model is chosen decisively and almost independently of the measurement times considered when cytoplasmic phosphorylated STAT1, in monomeric or dimeric form, or two forms of the receptor complex (IFN_R_JAKPhos_2 and IFN_R_JAK) are measured. The same is true of the complex model for measurements of two other forms of the receptor complex (IFN_R_JAK2 and IFN_R_JAKPhos_2_SHP_2). Otherwise the model selection outcome is time dependant or the choice of species is found to be uninformative.

**Figure 8:**
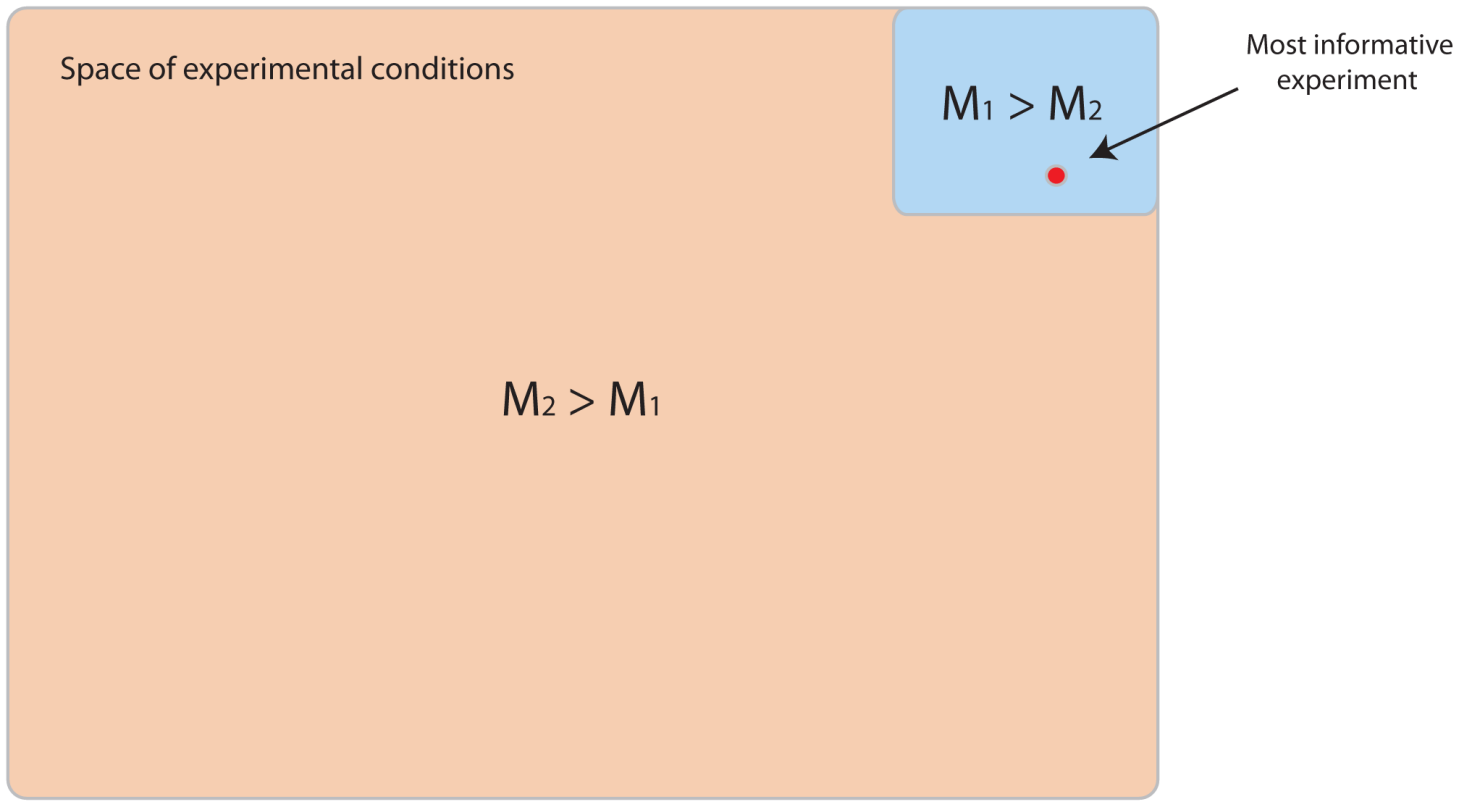
Model predictive power v.s. predicted confidence. Model 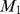 explains data produced from experiments in the blue region better than model 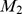. The opposite is true for the larger orange region. In this example, the most informative experiment generates data that favours model 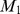. Performing model selection using such data will lead to the highest possible confidence we can generate for either model, and yet the chosen model will be the least predictive i.e. 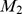 reflects reality better for the majority of considered experimental conditions. In this particular case, we have a greater chance of choosing the most predictive model by performing a random experiment.

Both these case studies make it clear that under the realistic assumption that all models are more or less incorrect, model selection outcomes can be sensitive to the choice of experiment. This observation has particular importance for studies that treat models as competing hypotheses that are decided between using experimental data; it is quite possible that if different experiments are undertaken, the conclusions drawn will also be different. In particular, the confidence calculated for such a conclusion (using the Jeffreys scale or another measure) can be misleading as a guide to how correct or predictive a model is (Figure 8a); in both the examples studied here, conditions exist such that any of the competing models can score a 'decisive' selection. The model selection outcome and associated confidence must therefore be strictly interpreted, as only increasing the odds of one model (with respect to others) for the data gathered under the specific experimental conditions.

In light of this observation, the role of experimental design may need to be examined further. Since different models can be selected depending on the experiment undertaken, the use of experimental design will necessarily lead to choosing the model which, for some 'optimal' experiment, has the highest possible predicted level of confidence i.e. experimental design implicitly makes confidence a selection criterion. Is it misleading to claim high confidence in a model selection result when the models have been set up (by extensions to mimic the optimal experiment) for this purpose? Is a bias introduced into the inference via experiment design? In the context of experiment design for parameter estimation, MacKay suggests this is not a problem [17], stating that Bayesian inference depends only on the data collected, and not on other data that could have been gathered but was not. Our situation here is different since we consider changes not only to the data collection procedure, but also the data generation process and in turn the competing models themselves. It seems plausible that some models will gain or lose more flexibility than others with regards to fitting data for a particular choice of experiment. Even if the actual model selection is not biased, the confidence we associate with it will scale with the optimality of the experiment. After performing the optimal experiment, should there be any surprise that the selected model seems to have high support from the data? We feel these questions need further investigation.

### Measuring sensitivity to model inaccuracies

In practical terms, the important question seems to be: how wrong does the model structure (or parameter values) have to be before the less predictive model (or that which captures less about the true system) is chosen? Clearly the answer is sensitive to the system and models under study, and moreover, the issue of how to compare the size of different structural inaccuracies is non trivial. Here, as a first attempt, we limit ourselves to considering the simple case of parameter inaccuracies in linear ODE models.

We define a 'base' model as the linear ode system defined by its Jacobian matrix with entries, 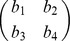
and 'extensions' to this model as an extra row and column, 
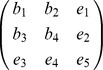



Biologically such an extension may represent the inclusion of an extra molecular species into the model, along with rules for how it interacts with components of the original system. Defining true base and extension models by 
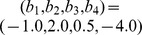
 and 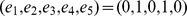, we consider two models, 
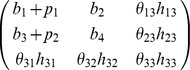
and 
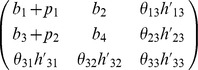
where 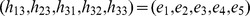 and 
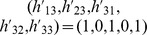
, are competing (true and false) hypotheses about the structure of the model extension, with a zero 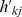 or 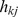 indicating a belief that species 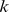 does not directly affect the rate of increase of species 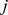. Parameters 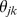, are the unknown strengths of these interactions, over which we place a 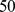 component mixture of Gaussians prior, fit to a uniform distribution over the interval 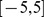 for each parameter. We represent inaccuracies in modelling the base as additive perturbations 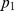 and 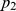. Data was generated by simulating the state of the first variable of the true model at times 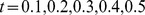, for initial condition 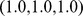.

**Figure 9:**
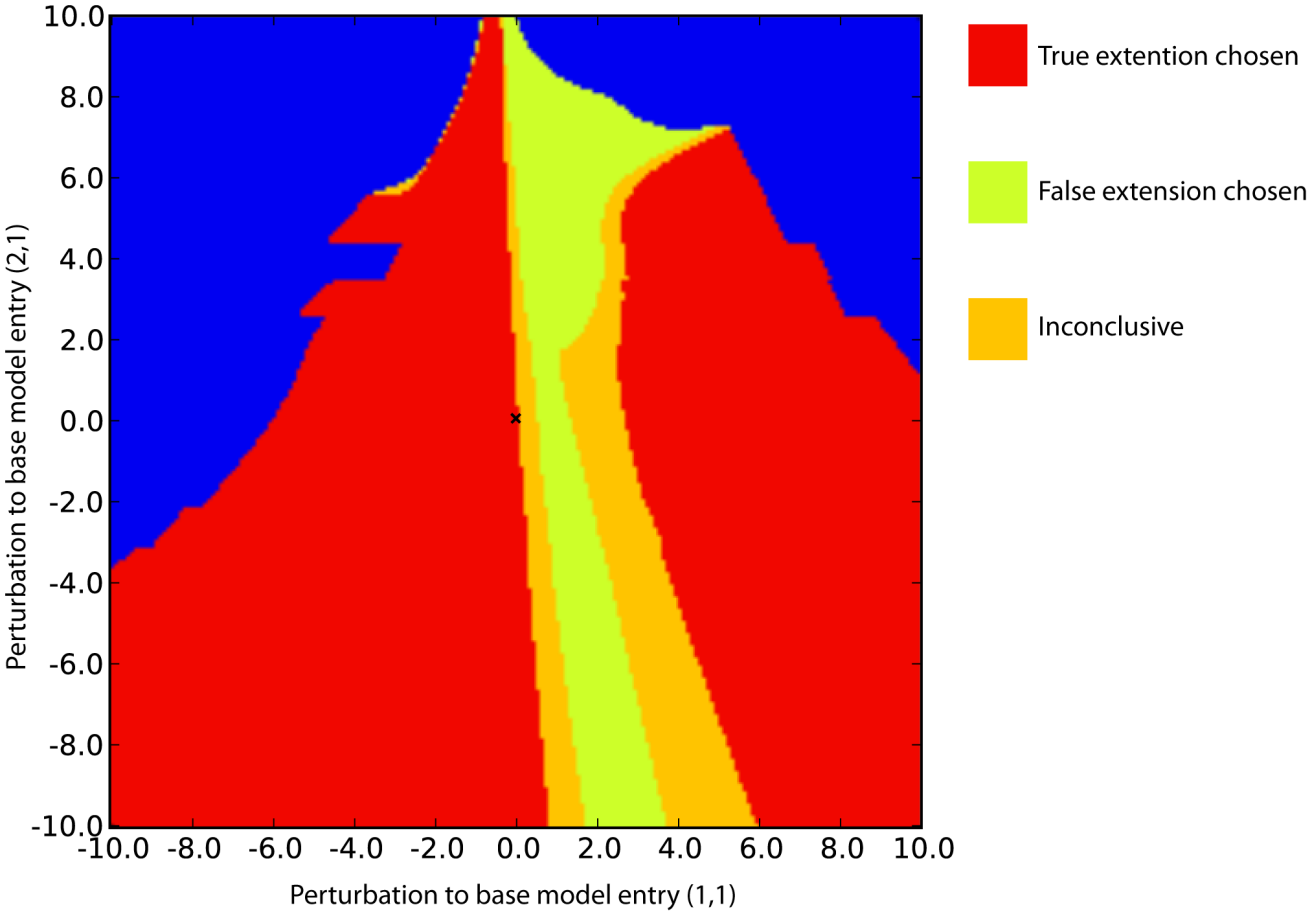
Model selection outcomes for 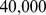, different pairs of linear ode models. Each model represents one of two competing hypotheses (the model extension), but with a different base model generated by perturbing Jacobian matrix entries 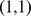 (x-axis) and 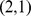 (y-axis). Regions where the different hypotheses receive support are given by the red (true extension), yellow (false extension), orange (no significant support for either extension), and blue (marginal likelihood values for both models are 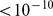) coloured regions. Increasing the threshold for the blue region to 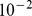 results in reduction of the red region, but not of the yellow. Using the true base model (represented by the cross at 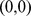), the true extension is also identified.

Model selection outcomes for 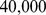 different pairs of values for the perturbations 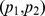, are shown in Figure 9. Distinct regions for each possible outcome are found and colour coded in the figure, with red indicating that the true extension has been identified successfully, yellow representing a decision in favour of the false extension, orange that evidence for either model is not substantial on the Jeffreys scale, and finally blue indicating that the marginal likelihood for both models is found to be less than 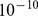, for which any conclusion would be subject to numerical error. Increasing this threshold has the effect of replacing red areas with blue.

In the majority of cases tested, the true extension is correctly identified despite inaccuracies in the base model. However, a set of perturbations are seen to confound the selection, and allow the false extension to obtain substantial support. Furthermore, the selection outcome is found to be more sensitive in some directions than others, with relatively small perturbations to base model entry 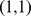 causing a change in outcome and creating decision boundaries near the lines 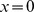 and 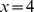. Prior to our analysis, it would be hard to predict these observations even when the true model is known and as simple as that explored here.

In real applications, where the true model is unknown and more complex, it may not be possible to tell whether a conclusion is an artefact of model inaccuracies, even when the truth of the conclusion itself can be tested by direct experimental measurement. However, the type of analysis undertaken here at least gives a measure of robustness for the conclusion to a range of model inaccuracies. Unfortunately, this remains difficult to implement in a more general setting – for example, in climatology, where the accepted method of coping with structural uncertainty is through the use of large ensembles of similar models produced by various research groups [18], a luxury that cannot be afforded on the scale of the most ambitious systems biology projects. While the practical challenges of dealing with large numbers of models is somewhat overcome by the model selection algorithm described above, a harder conceptual problem exists of how to define perturbations to more complicated classes of model, and to compare their strengths.

Finally, the example also highlights the difficulty of testing a hypothesis that represents only part of a model. The study shows that the implicit assumption that the base model is accurate, is not necessarily benign, and can affect any conclusions drawn – a result that is borne out by the logical principle that from a false statement, anything is provable.

## Discussion

The scale of the analyses detailed above, comprising thousands of marginal likelihood computations, requires extreme computational efficiency. Indeed it is completely beyond Monte Carlo based methods such as that recently developed by Liepe et al. [2], which are limited to exploring small sets of models and experiments. Here, the efficiency was obtained by using the unscented transform for propagating Gaussian mixture distributions through non-linear functions. Further computational savings can be made by exploiting the highly parallelizable nature of Flassig and Sundmacher's method [9], which we have extended for use with mixture distributed priors and stochastic state space models.

This efficiency has allowed us to explore model selection problems involving relatively large numbers of models and experiments, and investigate the robustness of model selection results to both changes in experimental conditions and inaccuracies in the models. Results from the latter two studies illustrate some common, but often ignored, pitfalls associated with modelling and inference. Firstly, we show that the conclusions of a model selection analysis can change depending on the experiment undertaken. Related to this, we observe that confidence in such a conclusion is not a good estimator of the predictive power of a model, or the correctness of the model structure. Further we note that the use of experimental design in this context maximises the expected discriminatory information available, and implicitly makes confidence in the outcome a criterion for model selection. In the future we intend to investigate the desirability of this property and how it affects the interpretation of the confidence associated with model selection outcomes.

At the heart of these issues is a lack of understanding of the implications of model (or parameter) inaccuracies. Often improved fits to data or better model predictions are interpreted as evidence that more about the true system is being captured. This assumption underlines a guiding paradigm of systems biology [19], where a modelling project is ideally meant to be a cycle of model prediction, experimental testing and subsequent data inspired model/parameter improvement. However, it is possible that improved data fitting and predictive power (although desirable in their own right) can be achieved by including more inaccuracies in the model. In the context of parameter estimation, this concept of local optima is widely known, and their avoidance is a challenge when performing any non-trivial inference. One simple method to do so is to include random perturbations in the inference, in order to 'kick' the search out of a local optimum. Perhaps a similar strategy might be included in the modelling paradigm; by performing random experiments, or adding or removing interactions in a model structure, data might be gathered or hypotheses generated that allows a leap to be made to a more optimal solution.

While we have been concerned solely with the statistical setting, it is reasonable to expect similar results can be found for alternative model discrimination approaches e.g the use of Semidefinite programming to establish lower bounds on the discrepancy between candidate models and data [20]. Here the particular subset of models that are invalidated will be dependent upon the experiment undertaken. However, emphasis on invalidating wrong models instead of evaluating the relative support for each at least reduces the temptation for extrapolated and, perhaps, false conclusions.

George E. P. Box famously stated that 'Essentially, all models are wrong, but some are useful'. Here we would add that if nothing else, models provide a natural setting for mathematicians, engineers and physicists to explore biological problems, exercise their own intuitions, apply theoretical techniques, and ultimately generate novel hypotheses. Whether the hypotheses are correct or not, the necessary experimental checking will reveal more about the biology.

## Materials and Methods

### The unscented transform

The UT is a method that describes how the moments of a random variable, 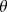, are transformed by a non-linear function, 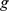. The algorithm begins by calculating a set of weighted particles (called sigma-points) with the same sample moments up to a desired order as the distribution 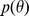. For the results shown here, we use a scaled sigma-point set 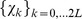 that captures both means and covariances [21], 
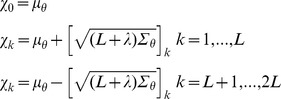
where 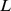 is the dimension of 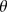, 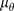 and 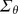 are the mean and covariance of 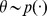, 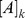 represents the 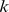
th column of a matrix 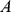, and 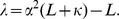



The sigma-point weights 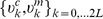 are given by, 
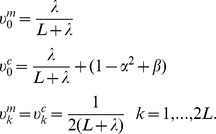
and finally, the parameters 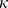, 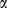 and 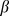 may be chosen to control the positive definiteness of covariance matrices, spread of the sigma-points, and error in the kurtosis respectively. For the results in this article we take 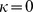 as is standard in the literature [22], and 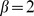 which is optimal for Gaussian input distributions, while 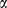, controlling the spread of sigma-points is taken small as 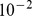 to avoid straddling non-local non-linear effects with a single Gaussian component [21].

The mean and covariance of the variable 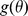, can be estimated as the weighted mean and covariance of the propagated sigma-points, 
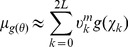
(4)

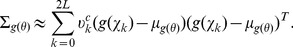
(5)


We denote the resulting approximate probability density function for 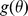, by 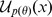.

By matching terms in the Taylor expansions of the estimated and true values of these moments, it can be shown that the UT is accurate to second order in the expansion. More generally, if the sigma-point set approximates the moments of 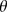 up to the 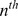 order then the estimates of the mean and covariance of 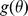 will be accurate up to the 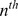 term [11]. Crucially, the number of points required (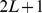 for this scheme) is much smaller than the number required to reach convergence with Monte-Carlo methods.

### Unscented model selection

We will consider discrete time state space models, 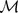, with state–transition (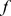) and observation (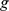) functions both parametrized by 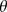,
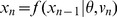
(6)

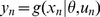
(7)where 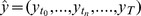, is the time series of 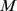 dimensional measurements that we are trying to model, 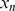 is the 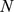 dimensional true state of the system at time 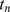, and 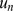, and 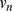 are independent, but not necessarily additive, Gaussian white-noise process and measurement terms. Bayesian model selection compares competing models, 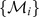, by combining the *a priori* belief in each model, encoded by the model prior distribution 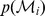, with the evidence for each model in the data 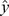, as quantified by the marginal likelihood,
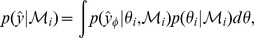
where 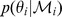 is the parameter prior for model 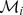. In the Bayesian setting, the relative suitabilities of a pair of models 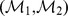 are often compared using the ratio of posterior probabilities, known as the Bayes factor,
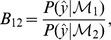
with a Bayes factor of 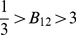 seen as substantial [23]. However, for complex or stochastic models, the marginal likelihood can be intractable, and so approximate likelihood free methods, such as Approximate Bayesian Computation are becoming increasingly important and popular within the biosciences [24]. A big drawback of such Monte-Carlo based algorithms is the large number of simulations – and associated computational cost – required to estimate the posterior distributions or Bayes factors. Even with GPU implementation [25], applications are currently still limited to comparing pairs or handfuls of models.

In order to address the issues raised above, a higher-throughput model selection algorithm is needed. Our approach will be to fit mixture of Gaussian models to the prior parameter distribution for each model, 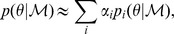
so that we can exploit the UT within the state-space framework to drastically reduce the number of simulations necessary to estimate the distribution of the output of the model. Gaussian mixture measurement and process noise can also be considered, as in the work on Gaussian sum filters [26], [27], although the number of mixture components required to model the output at each time point then increases exponentially, and in the case of long time series, component reduction schemes need to be implemented.

With this approximation, the marginal likelihood may be expressed as the sum, 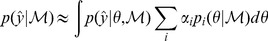
(8)

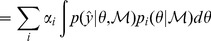
(9)

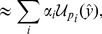
(10)where the components, 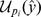, can be determined using the UT as described below. Note that the accuracy of the approximation can be controlled by the number of components used. However, in the presence of nonlinearities, choosing the number and position of components solely to fit the prior distribution may not be adequate. This is because we need to have enough flexibility to also fit a complex and possibly multi-modal output. Indeed, except at the asymptotic limit of dense coverage by the mixture components, it is possible to construct badly behaved mappings that will lead to loss of performance. For the applications visited in this article, the models proved well behaved enough such that a single component and 10 components respectively for the crosstalk and JAK-STAT systems sufficed for sufficient agreement with the nested sampling and Monte Carlo results. An improvement to the method described here would be to update the number of components automatically with respect to the model behaviour in a manner similar to how Gaussian mixtures can be adaptively chosen in particle based simulation of Liouville-type equations [28], [29].

For the deterministic case including the examples considered in this article, we have 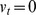, and the state–space model simplifies to, 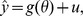
where might represent the simulation of certain variables of a system of ODEs, parameterised by 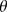, with additive measurement error 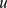. In this case the marginal likelihood can then be expressed as, 
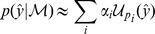
where each component 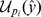 is obtained simply through application of the UT with input distribution 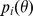, and liklihood that is Gaussian with mean, 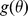, and variance, 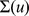.

To estimate the marginal likelihood in the stochastic case (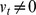), we assume the observation function takes the form of a linear transformation of the true state and measurement noise at time 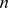 with additive noise, 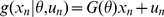
(11)where 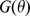 is an 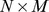 matrix. In practice this might correspond to the common situation where observations are scaled measurements of the abundance of various homo- or heterogeneous groups of molecules.

We may then write the mean of the observation, 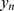, in terms of the statistics of 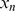, 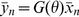
(12)for any 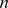, and from the bilinearity of the covariance function, the covariance between any pair of observations, 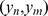, as, 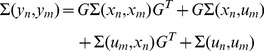
(13)




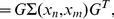
(14)since 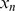 is independent of 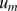 for all 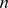 and 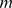. We now need to find expressions for the process state covariance terms in equation 14. To do so we apply the UT iteratively for 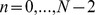 to transform the state-variable, 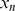 through the state-transition function 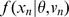, with input distribution 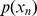 given by, 
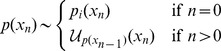



The result is a Gaussian approximation to the joint distribution 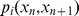 for each 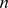, and hence also to the conditional distributions 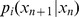. Given that 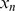 is a Markov process and that the product of Gaussian functions is Gaussian, we also have a Gaussian expression for the joint distribution, 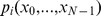, 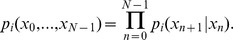



The covariance between any pair of observations 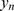 and 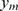, may then be found by substituting relevant entries from the covariance matrix of the density of Equation into Equation 14. The subsequent Gaussian approximation to the joint distribution of 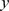, given 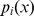, constitutes one component in the mixture approximation of the marginal likelihood given in Equation 10.

### Experimental design

We first introduce a vector of experiment parameters, 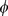, that describes how the dataset is created, specifying, for example, the times at which the system is stimulated, the strengths and targets of the stimuli, knockouts or knockdowns, along with the choice of observable to be measured at each time point. We can then model the system and experiments jointly, extending the 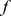 to include terms describing the possible experimental perturbations, and the 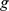 to capture the measurement options, 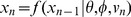
(15)

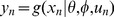
(16)


We assume that there is overlap between the system observables appearing in each model so that experiments that allow model comparison can be designed.

To illustrate how this might be done in practice, we consider a typical set of ordinary differential equations used to describe a gene regulatory mechanism, 
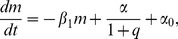
(17)

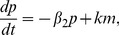
(18)where 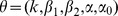 are the parameters controlling the rates of production and degradation of an mRNA, 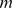, and a protein, 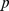, subject to the concentration of a repressor protein, 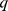. We define the state transition function 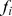 as their solution evaluated at the next measurement time-point 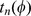 which is now dependant on the choice of 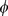, given the state at time 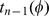, and subject to some additive noise 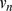. These equations have be extended as, 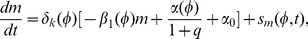
(19)

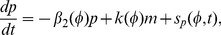
(20)to model a range of possible experimental perturbations, e.g. setting 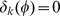 mimics a knockout of the gene producing mRNA 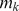, and 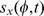 an input stimulus to species 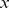. The observation function 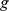, as before can be some linear function of the states, however, the selection of variables and coefficients is now an experimental choice specified by 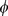, 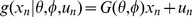



### Experimental design as an optimisation problem

Given a particular set of experimental options, 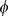, the marginal likelihood of model 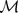 *for any possible* data set 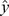 (the prior predictive distribution) can be estimated efficiently from equation 10, 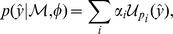
with the components 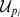 calculated with respect to the extended system and experiment model. Comparisons between such prior predictive distributions for competing models provides a means to predict the discriminatory value of a proposed experiment. Intuitively, values of 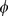, for which the prior predictive distributions of two models are separated, correspond to experimental conditions under which the models make distinct predictions of the system behaviour. Data gathered under these conditions are thus more likely to yield a significant model selection outcome. More formally, we can quantify the value of an experiment 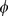, using the Hellinger distance between the prior predictive distributions, 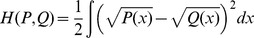
which takes the following closed form for multivariate Gaussian distributions, 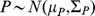 and 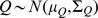, 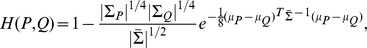
where, 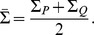
or for Gaussian mixtures, it can be evaluated using the method suggested in [30].

The experimental design problem may then be posed as an optimisation problem (the results in this article used a genetic algorithm [31] of population size 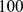 and 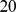 generations) over 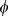 - we search for the set of experimental parameters, 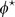, for which the Hellinger distance between the competing models 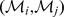, 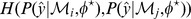, is maximal. 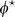 will then specify the experiment that gives the greatest chance of distinguishing between 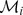 and 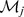. In the case where more than two models are considered, the cost function is taken as 
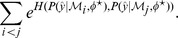
where the sum of exponentials is introduced to encourage selection of experiments with a high chance of distinguishing between a subset of the model pairs, over experiments with less decisive information for any pair of models, but perhaps a larger average Hellinger distance over all model pairs.
